# Supplementary material for: Evaluation of hip arthroscopy using a hip-specific distractor for the treatment of femoroacetabular impingement
Source: PLoS One. 2021 Feb 11;16(2):e0246655. doi: 10.1371/journal.pone.0246655 (PMC7877656; doi:10.1371/journal.pone.0246655)
Supplement: S1 Table — (DOCX) [file pone.0246655.s001.docx]

S1 Table.

| Hip | Sex | Side | TG | FAI Type | Interv time (min) | FAW (mm) | Supple-mentary Operative Gesture | AC CP | Complic-ations | HS (days) | Time to THA (years) | Sat | Do it again? | RTS |
| --- | --- | --- | --- | --- | --- | --- | --- | --- | --- | --- | --- | --- | --- | --- |
| 1 | M | L | 2 | Pincer | 225 | 14 | LD | Grade 2 | 0 | 2 | 2 | Yes | Yes | N/A |
| 2 | M | R | 0 | Pincer | 300 | MD | LD | Grade 2 | TO | 10 | N/A | MD | MD | MD |
| 3 | F | R | 0 | Mixte | 360 | MD | MD | MD | WB | 4 | 6 | Yes | Yes | N/A |
| 4 | F | L | 0 | Mixte | 270 | 16 | LD | Grade 0 | 0 | 2 | N/A | Yes | Yes | N/A |
| 5 | M | L | 0 | Mixte | 360 | 16 | LD, ACD | Grade 3 | 0 | 2 | N/A | Yes | Yes | EL |
| 6 | M | L | 0 | CAM | 240 | 12 | LD | Grade 1 | 0 | 3 | N/A | N | N | N/A |
| 7 | M | R | 1 | CAM | 330 | 16 | LD | Garde 0 | 0 | 2 | N/A | MD | MD | N/A |
| 8 | F | L | 1 | CAM | 255 | 16 | LD | Grade 4 | 0 | 3 | N/A | MD | MD | N/A |
| 9 | F | R | 1 | CAM | 330 | 18 | LD | Grade 3 | 0 | 2 | N/A | No | No | N/A |
| 10 | M | L | 1 | CAM | 260 | 15 | LD | Grade 3 | 0 | 3 | 3 | No | No | N/A |
| 11 | F | L | 0 | CAM | 200 | 12 | LD, MP | Grade 4 | 0 | 2 | N/A | Yes | Yes | IL |
| 12 | M | R | 1 | Pincer | 245 | 16 | LD, ACD | Grade 3 | 0 | 3 | N/A | N | N | N/A |
| 13 | M | R | 1 | Mixte | 185 | MD | LD | Grade 0 | 0 | 6 | N/A | MD | MD | N/A |
| 14 | F | R | 0 | Mixte | 340 | 10 | LD | Grade 0 | 0 | 4 | 2 | Yes | Yes | N/A |
| 15 | M | R | 0 | CAM | 485 | 18 | LD, MP | Grade 3 | TO | 4 | N/A | Yes | Yes | EL |
| 16 | F | L | 0 | Mixte | 285 | 12 | LD | Grade 1 | 0 | 3 | N/A | Yes | Yes | N/A |
| 17 | F | R | 1 | Mixte | 245 | 16 | LD, ACD | Grade 3 | 0 | 4 | N/A | Yes | Yes | N/A |
| 18 | F | R | 0 | Mixte | 180 | 20 | LD | Grade 1 | 0 | 3 | N/A | Yes | Yes | N/A |
| 19 | F | R | 0 | Pincer | 220 | 10 | LD | Grade 2 | 0 | 3 | N/A | Yes | Yes | N/A |
| 20 | M | R | 0 | Mixte | 240 | 17 | LD, MP | Grade 4 | 0 | 3 | N/A | Yes | Yes | IL |
| 21 | F | L | 1 | Mixte | 240 | 12 | LD, MP | Grade 4 | 0 | 3 | 4 | Yes | Yes | N/A |
| 22 | M | L | 2 | Mixte | 270 | 16 | LD | Grade 4 | 0 | 3 | N/A | Yes | Yes | IL |
| 23 | F | L | 1 | CAM | 185 | 15 | LD, ACD | Grade 2 | 0 | 3 | N/A | Yes | Yes | N/A |
| 24 | M | R | 2 | Mixte | 200 | 8 | LD, ACD | Grade 3 | 0 | 3 | 2 | Yes | Yes | N/A |
| 25 | F | L | 2 | Mixte | 150 | 10 | LD, ACD | Grade 4 | HSL | 3 | < 1 | No | No | N/A |
| 26 | M | L | 0 | Mixte | 210 | 6 | LD, ACD | Grade 2 | 0 | 3 | N/A | Yes | Yes | EL |
| 27 | M | R | 0 | Mixte | 175 | 6 | LD, ACD | Grade 3 | 0 | 3 | N/A | Yes | Yes | EL |
| 28 | F | R | 0 | Mixte | 205 | 16 | LD | Grade 0 | 0 | 3 | N/A | Yes | Yes | EL |
| 29 | M | L | 2 | Mixte | 145 | 11 | 0 | Grade 4 | 0 | 3 | N/A | Yes | Yes | IL |
| 30 | M | L | 0 | CAM | 190 | 12 | LD | Grade 3 | 0 | 3 | N/A | Yes | Yes | IL |
| 31 | F | L | 0 | CAM | 250 | 6 | LD, ACD | Grade 3 | 0 | 4 | 1 | Yes | Yes | IL |
| 32 | F | R | 0 | Pincer | 190 | 15 | LD, ACD | Grade 3 | LL | 4 | N/A | Yes | Yes | N/A |
| 33 | M | R | 0 | CAM | 180 | 12 | LD | Grade 1 | 0 | 3 | N/A | Yes | Yes | N/A |
| 34 | F | L | 3 | CAM | 240 | 10 | MP | Grade 4 | 0 | 3 | N/A | MD | MD | N |
| 35 | M | L | 1 | CAM | 300 | 20 | LR | Grade 1 | 0 | 4 | N/A | Yes | Yes | IL |
| 36 | F | L | 0 | CAM | 336 | 14 | LD | Grade 0 | 0 | 2 | N/A | N | N | N/A |
| 37 | M | L | 0 | Mixte | 180 | 6 | LD, ACD | Grade 3 | 0 | 3 | N/A | Yes | Yes | EL |
| 38 | M | R | 1 | Mixte | 180 | 12 | LD, MP | Grade 4 | 0 | 3 | N/A | N | N | IL |
| 39 | M | L | 0 | CAM | 150 | 12 | LD, ACD | Grade 3 | 0 | 3 | N/A | N | N | IL |
| 40 | M | R | 0 | CAM | 180 | 10 | LR | Grade 4 | 0 | 3 | N/A | Yes | Yes | EL |
| 41 | F | R | 0 | CAM | 180 | 10 | LR, ACD | Grade 2 | 0 | 3 | N/A | Yes | Yes | N/A |
| 42 | F | R | 0 | CAM | 135 | 14 | LD | Grade 1 | 0 | 3 | N/A | Yes | Yes | IL |
| 43 | F | R | 0 | CAM | 150 | 10 | LD, MP | Grade 4 | 0 | 7 | N/A | N | N | N/A |
| 44 | F | L | 1 | Mixte | 210 | 12 | LR | Grade 0 | 0 | 3 | N/A | Yes | Yes | N/A |
| 45 | M | R | 2 | Mixte | 120 | 12 | LD, MP | Grade 3 | 0 | 3 | N/A | Yes | Yes | N/A |
| 46 | M | L | 1 | CAM | 180 | MD | LD, MP | Grade 4 | 0 | 3 | N/A | Yes | Yes | EL |
| 47 | M | R | 0 | CAM | 180 | 10 | LD, ACD | Grade 2 | 0 | 2 | N/A | Yes | Yes | EL |
| 48 | M | R | 0 | CAM | 180 | 18 | LR, ACD | Grade 3 | 0 | 3 | N/A | Yes | Yes | MD |
| 49 | F | L | 1 | CAM | 160 | 12 | LD, ACD | Grade 2 | 0 | 4 | N/A | Yes | Yes | MD |
| 50 | F | R | 0 | CAM | 150 | MD | LD | Grade 2 | 0 | 3 | N/A | Yes | Yes | IL |
| 51 | M | L | 1 | Mixte | 240 | 12 | LR | Grade 2 | CL | 3 | N/A | Yes | Yes | N/A |
| 52 | M | R | 1 | Mixte | 180 | 12 | LD | Grade 1 | 0 | 3 | 2 | N | N | N |
| 53 | F | L | MD | CAM | 180 | 18 | LD, ACD | Grade 2 | 0 | 3 | N/A | N | Yes | N/A |
| 54 | M | L | 0 | Mixte | 180 | 10 | LD | Grade 1 | 0 | 2 | N/A | Yes | Yes | N |
| 55 | M | R | 0 | CAM | 180 | 10 | LD, ACD | Grade 2 | 0 | 2 | N/A | Yes | Yes | IL |
| 56 | M | R | 0 | Mixte | 180 | 10 | LD, ACD | Grade 1 | 0 | 3 | N/A | Yes | Yes | N |

ACD = acetabular cartilage lesion debridement, AC CP = acetabular chondropathy, EL = equal sports level, F = female, FAI = Femoroacetabular impingement, FAW = femoroacetabular widening, HS = hospital stay, HSL = hip subluxation, IL = Inferior sports level, Interv = intervention, L = Left, LD = labral debridement, LR = labral repair, M= male, MD = missing data, MP = microperforations, N/A = not applicable, R = Right, RTS = return to sports, Sat = satisfaction, TG = Tönnis Grade, THA = total hip arthroplasty, TO= torso oedema, WB = wound bleeding. The column “Do it again?” represents the answers of the patients to question whether they would undergo the same surgical procedure again if they had to reconsider it.
