# Supplementary material for: Evaluation of hip arthroscopy using a hip-specific distractor for the treatment of femoroacetabular impingement
Source: PLoS One. 2021 Feb 11;16(2):e0246655. doi: 10.1371/journal.pone.0246655 (PMC7877656; doi:10.1371/journal.pone.0246655)
Supplement: Attachment — Submitted filename: PLOS paper.docx [file pone.0246655.s002.docx]

**Title - Advantages of hip arthroscopy using an external hip distractor for the treatment of femoro-acetabular impingement.**

Comment 1 – looks irrelevant to the hypothesis and goal

“The goal of this study was to evaluate the learning curve of hip arthroscopy using a hip-specific distractor and the complications related to this procedure.” line 70

Removal/replacement of the word advantage with evaluation will reflect the idea better.

The word “advantage” draws the attention towards more benefits than the conventional traction table, however this is not the case here. Its only an evaluation of this traction technique.

Comment 2 – I prefer sticking to one name for the distractor used; either “external hip distractor” used in title or “hip-specific distractor” used in the introduction

**Abstract**

Comment 1 – word “per” should be replaced with peri

Comment 2 – poorly written. Needs to be re-written in a more attractive and informative way. Keeping in mind that this is the most read part of the paper. If not attractive, the reader will refrain from reading the paper.

**Introduction**

Generally, well-written covering the gap available in working with conventional traction table, and goal behind the study.

Comment 1 - Line 55: Distraction- of compression-type complications are related to the length of the……. Replace “of” with “and”

Comment 2 – I prefer sticking to one name for the distractor used; either “external hip distractor” used in title or “hip-specific distractor” used in the introduction

**Materials and Methods**

Generally, the materials and methods section lack the most important part the readers will be waiting for which is a precise and complete description of this specific distractor. Nothing was mentioned about the distractor apart form an intraoperative photo (Figure 3).

This is a major defect in this section that should be properly addressed under a specific subtitle together with proper illustrative photos or diagrams explaining its application and biomechanics.

Points of strength

- Type of study = retrospective September 2007 to March 2015
- Single disease = FAI
- Single Surgeon

Comment 1 – no reference was there for the preoperative conservative treatment protocol. On which basis this, 6m for athletes and 1-year conservative treatment protocols were set?

Comment 2 – Special mechanical analysis and attention should be paid and mentioned for the biomechanics of this distractor

? Material

? Line of forces pull, and Maximum force withstand

? Method of distraction in this instrument? Is it manual?

? Others, to be provided by manufacturer

Comment 3 – Lines 113,114 “Then, the peripheral compartment is assessed, and osteoplasty 113 of the femoral head-neck 114 junction is performed in cases of CAM-type lesions.”

I understood that you start working on the PC while traction is still applied… what is the advantage for this?

Comment 4 - Nothing was mentioned about the exclusion criteria or contraindication of using this distractor. Do you still use it in all patients? Even in osteopenic or osteoporotic bone?

Comment 5 – Supra- acetabular area is the pathway of the superior gluteal bundle supplying the gluteus medius, minimus and tensor facia lata muscles…. Did you have any injury for these important structures? I can see none in the results section…. Please describe your insertion technique to avoid these structures.

**Results**

Poor numbers for proper evaluation

- 56 hips were performed in 54 patients in 8 years
- 23 hips with OA
- 6 patients lost at follow up

Comment 1 – Line 154: Mean femoro-acetabular widening was 12mm [6 to 20].

The method of measurement was not described and verified in the M and M section. Please mention?

Comment 2 – Line 155: In all cases the hip was found to be sufficient distracted to be able to perform hip arthroscopy

Please verify and relate the distraction distances in different tonnis grades and different Genders?

Theses are the two main factors that affect the distraction power, these needs special results analysis and correlation.

Comment 3 - Complication and total hip arthroplasty conversion rates

This section is a critical section as its will be of main concern to the readers to understand the complications expected from this distractor.

1. I do not understand the major complication of the “hip subluxation”

Did it happen immediately postoperative or later? What was the expected reason? and why rapid conversion to THA? Were other treatment methods consumed?

I can see in the discussion section a brief analysis of this complication, however, no exact reason was detected or explained.

If available, please provide the postoperative radiology for this case, to share this abnormal complication with the readers.

2. There is no need to repeat the complications twice in paragraph and table format.
3. There is no need to mention the zero complications in the table format

**Discussion**

General unaddressed issue: as mention traction results into distraction and compression type problems. I can understand that this distractor avoids the compression problems, however, you mentioned that it avoids both despite using it without time limits!!!

Line 256- In our series we 256 encountered no traction-related neurovascular complications.

Line 63 could help beginner surgeons in performing 63 hip arthroscopy without concerning about the surgical time and thus traction-related 64 complications [1, 11].

I agree that traction here is focused on the hip as mentioned in the discussion but is this enough to avoid all the NV complication for distraction for anytime limit. I think time limit is still a very important factor and should never be underestimated. Please verify and discuss further….

The discussion is lacking the biomechanical values of the distractor. For example, the lines of pull it provides. We know that you need a lateral and distal line of pulls for the best hip asses and this is provided by putting the perineal post slightly laterally (not in midline) and adducting the hip to provide lateralization force with leg traction.

Can you explain and discuss similar pulling forces by the distractor and advantage/ disadvantage of it?

The discussion is focused on the surgeon learning curve, which I can see is not related to the device used. Please revise your discussion and focus it on the device used, which is the main issue here, explaining and discussing all aspects of its use.
