# Supplementary material for: Evaluation of hip arthroscopy using a hip-specific distractor for the treatment of femoroacetabular impingement
Source: PLoS One. 2021 Feb 11;16(2):e0246655. doi: 10.1371/journal.pone.0246655 (PMC7877656; doi:10.1371/journal.pone.0246655)
Supplement: Attachment — Submitted filename: Rebuttal letter.docx [file pone.0246655.s003.docx]

Subject Letter:

Response to reviewers

Dear Editor and Members and Reviewers of the Editorial Board,

Dear Editor in chief and reviewers, we thank you for the attention you’ve given our manuscript so far and we hope the changes we made to it will be to your liking. We’ve carefully read all your comments and we do hope that following answers and changes will help to clarify your decision.

Journal Requirements: When submitting your revision, we need you to address these additional requirements.

1. Please ensure that your manuscript meets PLOS ONE's style requirements, including those for file naming. The PLOS ONE style templates can be found at <https://journals.plos.org/plosone/s/file?id=wjVg/PLOSOne_formatting_sample_main_body.pdf> and <https://journals.plos.org/plosone/s/file?id=ba62/PLOSOne_formatting_sample_title_authors_affiliations.pdf>
   a. ***Response:*** *We controlled and ensured that our manuscript meets PLOS ONE’s style requirements, including those for file naming.*
2. Please carefully proofread your manuscript for typographical errors. For example, in your methods section “We performed a retrospective analysis of all succesive patients …” should be “We performed a retrospective analysis of all successive patients.
   a. ***Response:*** *Our manuscript has been carefully proofread as asked for potential typographical errors.*
3. PLOS requires an ORCID iD for the corresponding author in Editorial Manager on papers submitted after December 6th, 2016. Please ensure that you have an ORCID iD and that it is validated in Editorial Manager. To do this, go to ‘Update my Information’ (in the upper left-hand corner of the main menu), and click on the Fetch/Validate link next to the ORCID field. This will take you to the ORCID site and allow you to create a new iD or authenticate a pre-existing iD in Editorial Manager. Please see the following video for instructions on linking an ORCID iD to your Editorial Manager account: <https://www.youtube.com/watch?v=_xcclfuvtxQ>
   a. ***Response:*** *The corresponding author (MJ) has validated his ORCID iD in the Editorial Management as requested*
4. Please include captions for your Supporting Information files at the end of your manuscript, and update any in-text citations to match accordingly. Please see our Supporting Information guidelines for more information: <http://journals.plos.org/plosone/s/supporting-information>.
   a. ***Response****: Caption for supporting file has been added.*

Reviewer #1: Study Hypothesis: the use of Hip distractor for Hip arthroscopy was not associated with traction or compression complications, it would facilitate the learning curve. The aim of the study was to evaluate the learning curve of hip arthroscopy using a hip-specific distractor and the complications related to this procedure

The authors provided a good introduction stressing the fact that the use of external hip distractor during hip arthroscopy has not been associated with any of the traction or compression type complications, that otherwise could be associated with the use of traction table, hence They hypothesized that the use of a distractor would relieve the surgeon from the burden of Time stress during traction And will allow him completing the whole planned procedure without fear of traction or compression type complications.
They conducted a retrospective analysis with inclusion of all cases treated from September 2007 to March 2015 In a single center by a single surgeon. The operative technique is adequately described. They mention clear inclusion criteria.


Discussion of specific areas for improvement

Major points:
1. although the primary objective of the study was clearly defined, The descriptive and statistical analysis of the learning curve was inadequate. the construction of such learning curve, how and why it was grouped into those unequal 5 groups is not clear in the text and was only mentioned in the figure 4 caption

***Response:*** *We adjusted the descriptive of the analysis of the learning curve as requested in the section material and methods.*

*We adjusted also the part in material & methods describing the creation of the learning curves, but we didn’t perform any tests to evaluate changes as Hoppe et al stated in their systematic review that “Conceptually, a learning curve should be identified as a graph in which a consecutive number of cases are presented on the horizontal axis and some measure of proficiency or learning is presented on the vertical axis” (sic)* [*http://dx.doi.org/10.1016/j.arthro.2013.11.012*](http://dx.doi.org/10.1016/j.arthro.2013.11.012)*; Also testing between groups of 10 patients each might not clinically nor statically have a lot of meaning in our opinion.*

2. Another major point is the lack of description of any statistical method or test used to evaluate changes in the learning curve

***Response****:* *Also testing between groups of 10 patients each might not clinically nor statically have a lot of meaning in our opinion. (see response to previous point)*

Other points to be considered

1. In the methodology, there is no mention of exclusion criteria. Were there any?

***Response****: No, there were no exclusion criteria, except for a patient’s possible refusal to participate to this study. A learning curve is the analysis of all successive patients who underwent a specific surgery for a specific indication, if there were other exclusion criteria, it might not reflect a proper learning curve. For example, if we had to exclude patients of older age, we might have had to exclude some of the first patients which would not reflect the actual learning of a surgeon in this specific setting.*

2. In the section of outcome measurement, no functional scores were collected perioperatively to objectively measure the outcome and only patient satisfaction (subjective) and total hip conversion rate were collected.( questionable value with short follow up which in some cases as short as one year)

***Response****: Most learning curves limit themselves in analysing operative time and perioperative and postoperative complications. We actually also evaluated patient’s satisfaction rates and conversion/revision rates, which are parameters rarely evaluated when evaluating learning curves. The strong point of this study is that it actually evaluates the learning of hip arthroscopy for one specific indication, as some authors suggest that every gesture in hip arthroscopy might present with its personal learning curve. Indications are not mixed in this study.*

3. The authors describe That all cases head preoperative imaging studies (X-rays, CT and MRI) however there is no reporting of the radiological parameters commonly used to measure The cam and pincer deformities whether pre- or postoperatively to control the adequacy of the performed surgery. This is important to support the claim of completeness of the surgical gesture in all the cases.

***Response:*** *In the section results (subtitle general characteristics) preoperative radiological evaluation of all patients has been described in general. The claim of completeness is not in accordance of the whole CAM or the Whole pincer that has been corrected; I don’t think that anyone can claim that as easy. We claim that we could do all we planned to do, without concerning about time and traction-related complications. We were able to correct deformities, treat labral lesion and chondral lesion and control the absence of residual impingement with dynamic testing under fluoroscopic and arthroscopic visualisation.*

4. In the statistics section of the methodology, there is only mention of the used statistics software.
There is no mention of any statistical method or statistical test performed . Also whether the mentioned differences in the learning curve for intervention time and patient satisfaction were statistically significant or not.

***Response:*** *We adjusted the description of the statistics used in this manuscript.*

5. also In the methodology, why selection was limited to the 56 hips although the authors described that until currently the learning curve is still ever changing. Was there power calculation for this sample size determined?

***Response:*** *No there was no power calculated for this study. When starting the study, we had only those first 56 patients who presented with at least 1 year of follow-up. We could not include others as follow-up was less than 12 months.*

6. Line 111: labral reinsertion is performed after osteoplasty not vice versa.

***Response:*** *Line 111: you are correct. We changed this; sorry for this mistake in the manuscript, we must have overlooked it*

7. The number of labral reinsertions in relation to the number of pincer/ mixed impingement treated is relatively low, here one would like to know where these more advanced time consuming reinsertions are located in the learning curve.

***Response:*** *Where are those more time-consuming labral lesions situated in the learning curve? A very interesting question indeed. This has been added in the result section as those labral repairs were performed as from the 35^th^ case and not earlier, thus slightly increasing mean intervention in the learning curve. I also took the liberty to add a note in the discussion regarding this.*

8. line 191: patients younger than 35, how many are they? A more detailed age description for example with the median value should be included.

***Response****: Line 191: has been addressed in the revised manuscript.*

9. line 192, 193 reconsider this sentence.
***Response****: Line 192: has been rewritten.*

10. Concerning the 17% total hip arthroplasty conversion (9 hips) which was explained by the older age, here again the age of those patients should be mentioned (range and mean)
Secondly The cartilage condition intra operatively (grade and extent of cartilage damage) was not described this might be directly related.
Where in the learning curve are those cases located? persistence of symptoms, dissatisfaction and eventually total hip replacement could also occur in the beginning of learning curve, the adequacy of resections as mentioned before should be proven by radiological measurements?

***Response****: This has been addressed in the revised manuscript.*

Reviewer #2: Please review attached Doc in which I wrote all my requested changes
Thank you

Title - Advantages of hip arthroscopy using an external hip distractor for the treatment of femoro-acetabular impingement.
Comment 1 – looks irrelevant to the hypothesis and goal
“The goal of this study was to evaluate the learning curve of hip arthroscopy using a hip-specific distractor and the complications related to this procedure.” line 70
Removal/replacement of the word advantage with evaluation will reflect the idea better.
The word “advantage” draws the attention towards more benefits than the conventional traction table, however this is not the case here. Its only an evaluation of this traction technique.

***Response:*** *Title has been changed according as requested.*

Comment 2 – I prefer sticking to one name for the distractor used; either “external hip distractor” used in title or “hip-specific distractor” used in the introduction

***Response:*** *As requested, we also paid attention and from now on, the only term referring to the device used is “hip-specific distractor”.*

Abstract
Comment 1 – word “per” should be replaced with peri

***Response****: This has been changed.*

Comment 2 – poorly written. Needs to be re-written in a more attractive and informative way. Keeping in mind that this is the most read part of the paper. If not attractive, the reader will refrain from reading the paper.

***Response:*** *I tried to make the abstract more attractive and hope you will prefer that way.*

Introduction
Generally, well-written covering the gap available in working with conventional traction table, and goal behind the study.
Comment 1 - Line 55: Distraction- of compression-type complications are related to the length of the……. Replace “of” with “and”

***Response:*** *Has been changed.*

Comment 2 – I prefer sticking to one name for the distractor used; either “external hip distractor” used in title or “hip-specific distractor” used in the introduction

***Response:*** *We changed every “external hip distractor” with the term “hip-specific distractor”, so we stuck to only 1 term referring to the device as requested.*

Materials and Methods
Generally, the materials and methods section lack the most important part the readers will be waiting for which is a precise and complete description of this specific distractor. Nothing was mentioned about the distractor apart form an intraoperative photo (Figure 3).
This is a major defect in this section that should be properly addressed under a specific subtitle together with proper illustrative photos or diagrams explaining its application and biomechanics.

***Response:*** *Regarding your comment of major defect in our section of material and methods…. I adjusted the introduction of the article, explaining a bit more about the line of work and how the hip-specific distractor works in a biomechanical way, so our hypothesis seems more concrete and better understandable for those who does not apply regularly this device. On the other hand, the main goal of this manuscript was not to describe the biomechanics of the hip-specific distractor but to describe a series (with learning curve) using the device. The biomechanics with diagrams as requested have already been described in other works published by other authors. (Sadri H. Techniques in hip arthroscopy and joint preservation surgery; Elsevier Saunders, 2011: pp 113-120)*

Points of strength
• Type of study = retrospective September 2007 to March 2015
• Single disease = FAI
• Single Surgeon

Comment 1 – no reference was there for the preoperative conservative treatment protocol. On which basis this, 6m for athletes and 1-year conservative treatment protocols were set?

***Response:*** *No, the duration of conservative treatment before planning hip arthroscopy is still debated in the literature and not very clear. We know that some patients respond to conservative treatment hence surgery is not always warranted. But athletes tend to wait less before surgery because they need to restart their sports activities and cannot wait 1 year for conservative treatment to be completed followed by at least 6 months of rehabilitation after arthroscopic surgery.*

Comment 2 – Special mechanical analysis and attention should be paid and mentioned for the biomechanics of this distractor
? Material
? Line of forces pull, and Maximum force withstand
? Method of distraction in this instrument? Is it manual?
? Others, to be provided by manufacturer

***Response:*** *See response to the same point raised above (I adjusted the introduction of the article, explaining a bit more about the line of work and how the hip-specific distractor works in a biomechanical way, so our hypothesis seems more concrete and better understandable for those who does not apply regularly this device. On the other hand the main goal of this manuscript was not to describe the biomechanics of the hip-specific distractor but to describe a series (with learning curve) using the device. The biomechanics with diagrams as requested have already been described in other works published by other authors.)*

Comment 3 – Lines 113,114 “Then, the peripheral compartment is assessed, and osteoplasty 113 of the femoral head-neck 114 junction is performed in cases of CAM-type lesions.”

***Response:*** *you are correct. We changed this; sorry for this mistake in the manuscript, we must have overlooked it.*

I understood that you start working on the PC while traction is still applied… what is the advantage for this?

***Response:*** *Distraction is progressively diminished when we start to work on the PC compartment. This has been adjusted.*

Comment 4 - Nothing was mentioned about the exclusion criteria or contraindication of using this distractor. Do you still use it in all patients? Even in osteopenic or osteoporotic bone?

***Response:*** *To answer your question, we still indeed use it in every case of hip arthroscopy we perform. We feel that the joint opening offered by the hip-specific distractor allows us to treat much better all lesions encountered in the joint. Nowadays, we can associate labral reinsertion with cartilage repair techniques with great ease, which we feel might be much more difficult without the hip-specific distractor. Since hip arthroscopy is mainly indicated in a young sportive population, osteoporotic bone is rarely a major concern.*

Comment 5 – Supra- acetabular area is the pathway of the superior gluteal bundle supplying the gluteus medius, minimus and tensor facia lata muscles…. Did you have any injury for these important structures? I can see none in the results section…. Please describe your insertion technique to avoid these structures.

***Response:*** *Avoiding soft tissue damage: Placement of the schanz screws after blunt dissection and positioning of the protecting sleeve in the correct position. Drilling is performed through the protecting sleeve followed by screw insertion. This has been adjusted and added in the material and method, subsection ‘surgical technique’.*


Results

Poor numbers for proper evaluation
• 56 hips were performed in 54 patients in 8 years
• 23 hips with OA
• 6 patients lost at follow up

Comment 1 – Line 154: Mean femoro-acetabular widening was 12mm [6 to 20].
The method of measurement was not described and verified in the M and M section. Please mention?

***Response:*** *The Schanz screws (diameter of 6mm) were used to scale fluoroscopic images in order to be able to measure femoro-acetabular joint opening. This has also been added in the material and methods to clarify our measurement methods.*

Comment 2 – Line 155: In all cases the hip was found to be sufficient distracted to be able to perform hip arthroscopy
Please verify and relate the distraction distances in different tonnis grades and different Genders?
Theses are the two main factors that affect the distraction power, these needs special results analysis and correlation.

***Response****: we reviewed those cases as asked and added this to the manuscript. But it is very disappointed as 4 of the 5 hips presented no signs of osteoarthritis and 4 of the hips were males.*

Comment 3 - Complication and total hip arthroplasty conversion rates
This section is a critical section as its will be of main concern to the readers to understand the complications expected from this distractor.
1. I do not understand the major complication of the “hip subluxation”
Did it happen immediately postoperative or later? What was the expected reason? and why rapid conversion to THA? Were other treatment methods consumed?
I can see in the discussion section a brief analysis of this complication, however, no exact reason was detected or explained.
If available, please provide the postoperative radiology for this case, to share this abnormal complication with the readers.

***Response:*** *this complication has been further clarified in the manuscript as asked. We did modify this section so complications were not repeated both in text and table as asked. And we removed the “zero” complications of the table as asked, although this was to highlight the absence of traction-related complications.*

2. There is no need to repeat the complications twice in paragraph and table format.

***Response:*** *This has been addressed in the revised manuscript.*

3. There is no need to mention the zero complications in the table format

***Response:*** *This has been addressed in the revised manuscript.*

Discussion

General unaddressed issue: as mention traction results into distraction and compression type problems. I can understand that this distractor avoids the compression problems, however, you mentioned that it avoids both despite using it without time limits!!!

Line 256- In our series we 256 encountered no traction-related neurovascular complications.
Line 63 could help beginner surgeons in performing 63 hip arthroscopy without concerning about the surgical time and thus traction-related 64 complications [1, 11].

I agree that traction here is focused on the hip as mentioned in the discussion but is this enough to avoid all the NV complication for distraction for anytime limit. I think time limit is still a very important factor and should never be underestimated. Please verify and discuss further….

***Response:*** *Regarding you concern about time limit and potential NV complications. Up to now, all series describing the use of a hip-specific distractor have never been associated with traction-related complications regardless of the intervention time as seen in series using an orthopaedic traction table. I admit, not a lot of surgeons do use a hip-specific distractor as some might think it less attractive due to its more invasiveness than an orthopaedic traction table, so there are not a lot of studies describing the use of hip-specific distractor, which is one of the reasons why this one might be very interesting. But its use has never been associated with traction-related complications regardless of the intervention time. I don’t think that this needs further description in the discussion section. Nevertheless, I do agree that time is a very important factor. A hip arthroscopy should not take a whole day to be performed, for the patient’s sake.*

The discussion is lacking the biomechanical values of the distractor. For example, the lines of pull it provides. We know that you need a lateral and distal line of pulls for the best hip asses and this is provided by putting the perineal post slightly laterally (not in midline) and adducting the hip to provide lateralization force with leg traction.
Can you explain and discuss similar pulling forces by the distractor and advantage/ disadvantage of it?

***Response:*** *This is a retrospective case series describing the use of the distractor. It is not a biomechanical study evaluating the feasibility of the hip-specific distractor. Others authors have already described this. (Also see previous response regarding similar comment.) But I don’t think that a text-book description of the biomechanics of the hip-specific distractor has its place in the discussion of this study.*

The discussion is focused on the surgeon learning curve, which I can see is not related to the device used. Please revise your discussion and focus it on the device used, which is the main issue here, explaining and discussing all aspects of its use.

***Response:*** *Personally, I don’t understand why we should revise the entire discussion, since this study is describing “a learning curve”, it might be normal that it is compared to other published learning curves in order to see if there might be potential advantages in using this device. Throughout the entire first part (1 page) of the discussion we discuss complications associated or not with the device, which is very important since avoidance of traction-related complication is one of the major reasons why a hip-specific distractor is so interesting. Lines 281 to 293 are dedicated to the pros and cons of the device. Repeating the description of the surgical technique has no necessity in the discussion in our opinion.*

Thank you for considering our manuscript,

Thank you for your revision and comments, they were very helpful.

Sincerely,

The authors,

Marc Jayankura & Tatiana Charles
