# Supplementary material for: Evaluation of hip arthroscopy using a hip-specific distractor for the treatment of femoroacetabular impingement
Source: PLoS One. 2021 Feb 11;16(2):e0246655. doi: 10.1371/journal.pone.0246655 (PMC7877656; doi:10.1371/journal.pone.0246655)
Supplement: Attachment — Submitted filename: rebuttal letter 2.docx [file pone.0246655.s004.docx]

Subject Letter:

Response to reviewers

Dear Editor and Members and Reviewers of the Editorial Board,

Dear Editor in chief and reviewers, we thank you for the attention you’ve given our manuscript so far and we hope the changes we made to it will be to your liking. We’ve carefully read all your comments and we do hope that following answers and changes will help to clarify your decision.

Response to Reviewer #1:

1- Response to point 1: the statement by Hoppe et al in their systematic review that “Conceptually, a learning curve should be identified as a graph in which a consecutive number of cases are presented on the horizontal axis and some measure of proficiency or learning is presented on the vertical axis” (sic) <http://dx.doi.org/10.1016/j.arthro.2013.11.012>; does not contradict a statistical significance testing. In the same article, a cut off of 30 cases could be identified; this could have been used in this study to make 2 bigger groups for testing instead of testing between groups of 10 patients each with little meaning.
   The authors in the current study describe already a 22% improvement in intervention time between the first 30 cases and the following cases. Is this difference statistically significant?

*Response: As requested we calculated whether or not this difference of intervention time between the first 30 cases and the last 26 cases was statistically significant. It was statistically significant with a p-value of 0.0002. Statistical tests applied and results were added in the material & methods section as well as in the result section.*

2- Again Concerning the 17% total hip arthroplasty conversion (9 hips) the authors in their revision gave more information about the age of this group, however, they did not mention how these cases plotted on the learning curve and again the cartilage condition intra operatively (grade and extent of cartilage damage) still needs to be described.
   Some of the important causes of total hip conversion could be either a high Tönnis grade of arthritis preoperative, extensive cartilage damage or inadequate FAI-correction or even over-correction which is common in the beginning of learning curve.
   where in the learning curve are those cases located was still not mentioned in the revised manuscript. Unfortunately, the authors could not provide a perioperative radiological evidence of adequate deformity correction. For example, a perioperative alpha angle correction and some of the acetabular measures as the CE angle should be provided.

*Response:* *In the result section describing THA conversion rates we also refer to table S1 with complementary results representing all cases performed (from the first case to the last). The table shows exactly where those cases of THA conversions are situated in the learning curve as well as the exact operative gestures performed during hip arthroscopy. We added a column resuming acetabular chondropathy graded during the intervention. We also added a few comments on the subject in the hope it clarifies this further for the readers in the discussion section.*


3- Minor spelling errors

• Line 30 correct to performed

*Response: This has been addressed in the revised version.*

• Line 53 Complications: remove S

*Response: This has been addressed in the revised version.*

• Line 96 proven FAI and failed conservative treatment where considered for an arthroscopic procedure: correct to were?

*Response: This has been addressed in the revised version.*

• Line 116 hip-specific external hip distractor: remove 2nd hip

*Response: This has been addressed in the revised version.*

• Lines 128, 129: Labral lesions are assessed and debridement if required. The sentence is not complete, correct to : Labral lesions are assessed and debridement was performed if required.

*Response: This has been addressed in the revised version.*

• Line 154 to analyse de learning curve: correct to the ?

*Response: This has been addressed in the revised version.*

Thank you for considering our manuscript,

Thank you for your revision and comments, they were very helpful.

Sincerely,

The authors,

Marc Jayankura & Tatiana Charles
