# Supplementary material for: Evaluation of hip arthroscopy using a hip-specific distractor for the treatment of femoroacetabular impingement
Source: PLoS One. 2021 Feb 11;16(2):e0246655. doi: 10.1371/journal.pone.0246655 (PMC7877656; doi:10.1371/journal.pone.0246655)
Supplement: Attachment — Submitted filename: rebuttal letter 3.docx [file pone.0246655.s006.docx]

Subject Letter:

Response to reviewers

Dear Editor and Members and Reviewers of the Editorial Board,

Dear Editor in chief and reviewers, we thank you for the attention you’ve given our manuscript so far and we hope the changes we made to it will be to your liking. We’ve carefully read all your comments and we do hope that following answers and changes will help to clarify your decision.

Response to Reviewer #1:

My single comment here has to do with the supplementary materials table. I had to guess that the (redo?) Column means if the patient would accept to have the surgery again when asked in the follow up. Is this correct. Please clarify this so the reader doesn't mistake this for revision rate or something.

*Response: Yes, you are correct indeed. We changed the name of the column from “Redo?” to “Do it again?” and added a comment to clarify this column in the legend below the table. We hope this will clarify this further for the readers.*

Thank you for considering our manuscript,

Thank you for your revision and comments, they were very helpful. It was a real pleasure and honour to collaborate with you for our manuscript.

Sincerely,

The authors,

Marc Jayankura & Tatiana Charles
